# Alternative Pathways in Astrobiology: Reviewing and Synthesizing Contingency and Non-Biomolecular Origins of Terrestrial and Extraterrestrial Life

**DOI:** 10.3390/life14091069

**Published:** 2024-08-27

**Authors:** Kuhan Chandru, Christian Potiszil, Tony Z. Jia

**Affiliations:** 1 Space Science Center (ANGKASA), Institute of Climate Change, National University of Malaysia, Selangor 43600, Malaysia; 2 Polymer Research Center (PORCE), Faculty of Science and Technology, National University of Malaysia, Selangor 43600, Malaysia; 3 Institute of Physical Chemistry, CENIDE, University of Duisburg-Essen, 45141 Essen, Germany; 4 The Pheasant Memorial Laboratory for Geochemistry and Cosmochemistry, Institute for Planetary Materials, Okayama University, Misasa 682-0193, Tottori, Japan; cpotiszil@okayama-u.ac.jp; 5 Blue Marble Space Institute of Science, Seattle, WA 98104, USA; 6 Earth-Life Science Institute, Tokyo Institute of Technology, Meguro-ku 152-8550, Tokyo, Japan

**Keywords:** origins of life, extraterrestrial life, contingency, non-biomolecules, agnostic biosignatures

## Abstract

The pursuit of understanding the origins of life (OoL) on and off Earth and the search for extraterrestrial life (ET) are central aspects of astrobiology. Despite the considerable efforts in both areas, more novel and multifaceted approaches are needed to address these profound questions with greater detail and with certainty. The complexity of the chemical milieu within ancient geological environments presents a diverse landscape where biomolecules and non-biomolecules interact. This interaction could lead to life as we know it, dominated by biomolecules, or to alternative forms of life where non-biomolecules could play a pivotal role. Such alternative forms of life could be found beyond Earth, i.e., on exoplanets and the moons of Jupiter and Saturn. Challenging the notion that all life, including ET life, must use the same building blocks as life on Earth, the concept of contingency—when expanded beyond its macroevolution interpretation—suggests that non-biomolecules may have played essential roles at the OoL. Here, we review the possible role of contingency and non-biomolecules at the OoL and synthesize a conceptual model formally linking contingency with non-biomolecular OoL theories. This model emphasizes the significance of considering the role of non-biomolecules both at the OoL on Earth or beyond, as well as their potential as agnostic biosignatures indicative of ET Life.

## 1. Introduction

“…*any replay of the tape (of life) would lead evolution down a pathway radically different from the road actually taken*…”

—Stephen Jay Gould, 1989

Contingency is associated with counterfactual thinking, which involves asking ‘what if’ questions. For example, one might ask, ‘Would event **B** have happened if event **A** had not occurred, and what would the alternative to event **B** be?’ Such questions can provide useful insights into understanding history [1], science [2], and even one’s personal life [3].

This exploration of counterfactual thinking alludes to Gould’s famous quote where contingency was introduced in evolution, i.e., how historical events (e.g., extinction events) shape evolutionary processes over very long timescales, a phenomenon known as macroevolution. The quote emphasized his perspective or reinterpretation of the Burgess Shale’s fossilized marine invertebrates. The fossils from the Cambrian period display a variety of *weird*-looking body plans, meaning unique structures and anatomical organizations unrelated to those of modern animals. Only a limited number of these basic body plans survived in all modern animals [4]. In other words, the critical survivors of extinction events of the Cambrian [5] went on to become the fauna as we know it today. He argues that without the extinction events, which wiped out most of the Cambrian fauna towards the end of the period, modern animal life would not look as it does today, thereby adding to the unpredictability of evolution [4].

**Figure 1:**
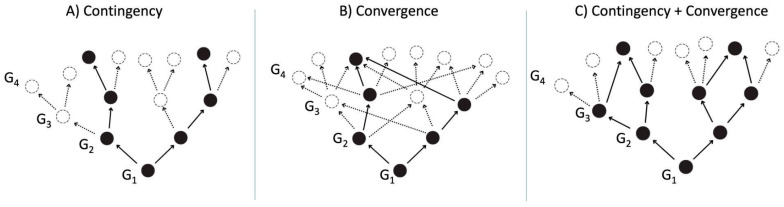
(**Panel A**) represents contingency outcomes starting from Generation 1 (G1) (a single point of origin) to G4. The two black circles at G4 indicate the ‘final’ destinations that have gone through different paths through G2 and G3, chosen from various possibilities, as a result of contingency occurring in one or more generations (dashed empty circles). On the other hand, (**Panel B**) illustrates a convergent outcome. Despite starting from a single point of origin at G1 and passing through different points at G2 and G3, the final outcome arrives at the single point (G4). In contrast, (**Panel C**) indicates a combination of contingency and convergence. Modified from [9].

Nevertheless, because of Gould’s large brush stroke description and often confusing take on contingency, many philosophers of science have tried to formally define what Gould meant by contingency on evolutionary outcomes [6,7,8,9]. The definitions can be primarily simplified in two ways. First, contingency is cause-dependent, meaning a sequence (or a dynamic web) of prior causative events governs a particular evolutionary outcome, making the outcome unpredictable [6]. Had these events transpired differently (or not happened at all), the outcome would have been altered. Second, contingency is the sensitive dependence of an evolutionary outcome on initial conditions [10]. An alternative outcome could emerge if minor changes occurred in initial conditions, such as geological, biochemical, genetic mutation, developmental, phylogenetic, and/or ecological factors. Figure 1A is a pictorial representation of Gouldian contingency’s variations. A thorough philosophical treatise of contingency is well discussed in [10].

Within the context of macroevolution, “contingency” contrasts with “determinism”. Generally, determinism is a philosophical principle that every event or state of affairs is the inevitable result of preceding events and conditions, governed by the laws of nature, leaving no room for alternative possibilities and, therefore, making outcomes predictable [11]. Or, more simply, different things tend to head towards a commonality or similarity over time. For example, evolutionary determinism is best demonstrated by convergence, i.e., the repeatable evolutionary outcome determined by natural selection [12,13]. Convergence is thought to be ubiquitous because it relies upon paleontological and biological data of both past and present animal life. It implies there are only a few ways for organisms to develop even though they may be coming from different genomic lineages, thus making evolution predictable. For example, bats, birds, and insects independently developed their flying ability [14]. Similarly, the development of flippers or paddles in present and past marine animals, such as ichthyosaurs, dolphins, penguins, seals, sharks, and turtles, indicates that these features were adapted to swim. Both examples demonstrate that there are only a few ways to fly and swim. Therefore, employing the ‘replay’ metaphor for convergence, if we were to replay the tape of life—even starting from different initial conditions—it would likely produce macroevolutionary outcomes that closely resemble life as we know it [10] (Figure 1B).

While these examples for convergence are sound, they do not necessarily justify the ubiquitousness of convergence, as there are equally compelling examples that signify contingency being ubiquitous, too. For instance, Gould used kangaroos (and marsupials in general) to illustrate contingent evolution. Their significant differences from herbivores worldwide, such as their distinctive hopping locomotion and unique reproductive method—where their babies do not develop within a placenta [15]—underscores their dominant presence within the Australian fauna. Why did convergence not influence the evolution of kangaroos towards the stereotypical herbivores? Similarly, the duck-billed platypus stands out as a mammal with no close relatives, showcasing another instance of contingency in evolutionary history. Other examples include the fauna of New Zealand—a land devoid of mammals, elephants, koalas, etc., indicating the outcomes from evolution are not ubiquitously convergent [16].

Despite the clear theoretical demarcation of contingency and convergence (shown above), it is very difficult to categorically determine which played a dominant role in the macroevolution of terrestrial life at various times during history, as both ideas are severely debated [17,18]. Convergence proponents [19] conclude that convergence outcomes are ubiquitous, whereas others believe they are far from ubiquitous [4,16]. It is also conceivable that contingency and convergence may have intersected at various historical points (Figure 1C), or a spectrum between contingency and convergence could also likely exist. For example, evolution could have started contingently (unpredictable). And, as time progressed and certain conditions or thresholds were met, the process became more predictable and less subject to random chance, transitioning to a more deterministic pattern. Nevertheless, the relationship between deterministic and contingent factors in evolutionary processes is indeed more nuanced than the binary distinction we have outlined here [10].

However, this debate between contingency and convergence can only be solved if (and, hopefully, when) data on extraterrestrial (ET) life is acquired. With such data, it would be possible to distinguish contingency from convergence (or vice versa) or determine whether there is a spectrum between them in macroevolution. Such data may include the habitat of ET life, its developmental biology features, genetics, mutations, chemical makeup, ecology, and its planet’s climate and geological characteristics. For instance, if ET life exhibits familiar body plans, this might suggest convergence as the driving force in macroevolution. Conversely, if the ET life’s body plans deviate from known Earth macrofauna, perhaps resembling past macrofauna like that of the Burgess Shale, then contingency could be seen to shape macroevolution based on Gould’s interpretation.

It is worth emphasizing that evolutionary contingency and determinism (demonstrated by convergence) are built upon the foundation of complex cellularity and beyond. However, what if one broadens these concepts to the origins of life (OoL) on and off Earth (OoL of ET Life) right before nascent cellularity?

For example, if cellular ET life uses similar biochemistries as terrestrial life, one can conjecture that “determinism” could be more prevalent than “contingency”. This would suggest that the biochemistry of terrestrial life is the *only* plausible form of life in the universe, regardless of the physical, chemical and environmental conditions that developed them (i.e., *N* = 1). On the other hand, if the discovered ET life operates using vastly different biochemistries—i.e., xeno-biochemistry that includes non-biomolecular compounds (see definition in Table 1)—we can argue that contingency may have played a role. This would indicate that ET life did not emerge in the same way as terrestrial biochemistry (i.e., *N* = 2, which suggests possibly that *N* ≥ 2). Finally, if ET life utilizes both contemporary biomolecules and some non-biomolecules within their makeup and biochemistry, then there could be an interplay (or a spectrum) of both determinism and contingency in shaping this ET life. Thus, it is necessary to consider the role of both contingency and determinism in astrobiology and, in particular, life detection, as life and/or the origins of life (OoL) on Earth may or may not be different from that on other possibly life-bearing planetary bodies, which could themselves have been potential bioreactors (e.g., [20,21,22,23]).

However, these scenarios also come with another required *Gedankenexperiment* (i.e., thought experiment), as ET life using one or more non-biomolecules could indicate that such non-biomolecules were a post-OoL recent invention of the organism itself rather than contingency. For example, even on present-day Earth, some bacteria use a genetic code incorporating additional non-canonical amino acids (i.e., the 21st and 22nd amino acids) with the canonical amino acids involved in their biochemistry [34]. Another scenario would be the discovery of ET life incorporating non-biomolecules, simply indicating that its evolution is still “in progress” and that this life will (eventually) lose the use of the non-biomolecules and result in a form identical to terrestrial life (i.e., “determinism” is still in progress) [35]. However, even if one can discount the plausibility of contingency in either *Gedankenexperiment* case, it is still true that, in both cases, at least one non-biomolecule plays or played a role at some point during the evolution of said life. Therefore, the detection of non-biomolecules extraterrestrially remains an important aspect of ET life detection.

In regards to the role of contingency vs. determinism during the OoL on Earth, the notion that biological evolution necessarily resulted *from* the prebiotic world leads to one investigative strategy, wherein the terrestrial OoL is viewed deterministically through the lens of present-day biology. This perspective posits that biomolecules were assembled to become the common ancestor [31]. Most terrestrial OoL theories are built upon the assumption that, because biomolecular building blocks are the mainstays of life and life relies on biomolecules to function, primitive life necessarily used the same or similar biomolecules, thereby illustrating a deterministic process resulting in a predictable outcome.

From this perspective, one can also conjecture that, because we employ a biomolecular mindset to test and investigate OoL theories, the same set of biomolecules has thus generally been applied to search for “biosignatures” indicating ET life [36]. Such a mindset adheres to the observer selection effect, i.e., a bias that arises when considering observations or data (e.g., life on Earth) from the perspective of an observer (modern humans) limited by their own existence or perspective. Hence, as described above, and in an attempt to reduce the observer selection effect, it is still plausible to consider that non-biomolecules could have participated in and driven the OoL before the emergence of biomolecules [30,37,38].

Conversely, if one assumes that contingency plays a role during the OoL (both on and off Earth), it can be argued that the (bio)molecules comprising ET life can also be, or perhaps are even likely to be, different than those comprising terrestrial life. This is because abiotic chemical space (see below for more details) within an ancient geological environment (on Earth, for example) is not limited to specific biological building blocks but is filled with many prebiotic chemical compounds, including some amount of the biological building blocks. This chemical space likely contained a minority of biomolecules and a majority of non-biomolecular compounds [30,39,40]. A major unanswered question is how biomolecules were “selected” from this large chemical space and organized/assembled to somehow lead to the emergence of life, i.e., chemical evolution. For example, perhaps the minority biomolecules each inherently contained some selective advantage over the majority non-biomolecules in primitive terrestrial geological conditions, although those biomolecules also must have necessarily possessed some method to accumulate and increase in concentration/amount [24]. However, in a different environment, such as another planetary body, even if we assume an identical or similar chemical space to that of primitive Earth (which itself might have been improbable or impossible, given the complexity of the primitive milieu), those selective advantages may not have existed for the “terrestrial” biomolecules in this different environment. Instead, in this different environment “terrestrial” non-biomolecules might have been selectively advantageous.

Or, perhaps, the composition of a hypothetical chemical space off Earth itself was different than that of Earth, where “terrestrial” biomolecules no longer exist at all (for example, they are degraded or cannot be synthesized in the first place). Thus, while the role of primitive biomolecules in the emergence of terrestrial life may be irrefutable, for the reasons above, one must also consider that life elsewhere may have taken a different chemical path that did not rely solely on biomolecules. Chemical evolution in a different planetary environment (e.g., [41,42]) could lead to an alternative version of life—life as we *don’t* know it—potentially involving different elements, unusual bonds, alternative biopolymers, metabolites, or even genetic or catalytic systems.

With the context mentioned above, we review the role of contingency in prebiotic chemistry and chemical evolution. In particular, we discuss the conjecture that contingency had a hand in the OoL, potentially leading to the emergence of prebiotic chemistries that incorporated non-biomolecules as essential components. However, since modern biology does not contain these non-biomolecules, it is possible that at some point in history, if these non-biomolecules did have a major role in the origins of life, they may have become biological vestiges that are no longer used. Such a concept of contingency may be feasibly applied to ET life detection as well, which, up until now, has generally been based exclusively on what is understood about biology and seeks to search for biosignatures directly related to modern biology. Here, we synthesize concepts from both contingency and non-biomolecular OoL theories to discuss the relevance of non-biomolecules as reasonable biosignatures to consider in life detection missions, as the role of contingency in prebiotic chemistry coupled with the complexity of the chemical milieu on any extraterrestrial surface suggests that such non-biomolecules could have played a role at some point in the development of ET life.

## 2. The *N* = 1 Problem and Its Relevance in the Search for Extraterrestrial Life

Many hypotheses can convincingly argue that a particular OoL narrative is correct. However, we will not discuss the details of such narratives, as this has been better reviewed elsewhere (e.g., [43,44]). For example, the RNA world theory [43] argues that a controlling or regulating entity for core biochemical processes was first featured in early life. Here, control or regulation began with the advent of self-replicating RNA that subsequently underwent Darwinian-type evolution to develop catalytic properties. This led to the co-evolution of peptides that could have catalyzed metabolic reactions, and then later, supported other processes essential to cellular life. Thus, according to this theory, Darwinian-type evolution is the *prelude* to the OoL, and its emergence was required to “kick-start” the OoL.

On the other hand, the “metabolism-first” theory [45] argues that the Darwinian-type evolution came much later. In this theory, the OoL began at the advent of a self-organizing network of prebiotic organosynthesis, i.e., interconnecting reaction networks of small molecules continuously exchanging molecules and energy with the (geo)environment, eventually resulting in assembly of higher-ordered structures. These higher-ordered structures include large functional molecules, such as RNA, as a *product* of the self-organizing network. Therefore, Darwinian-type evolution, which resulted from the large self-organized network originating from organosynthesis, was a product of the initial stages of OoL. However, regardless of whether metabolic cycles or self-replicating preceded Darwinian evolution, a genetic adapter that closely links genotype to phenotype could have been necessary. In fact, the evolution of such an adapter may have been a deterministic bottleneck, and a genetic adapter (like tRNA in the modern day) would have been necessary to afford efficient Darwinian evolution [46,47,48].

These (and other) OoL hypotheses are compelling in their own right, such as the peptide-RNA-metabolism-lipid world [49]. However, all require further exploration due to unsolved problems. For example, all current OoL hypotheses rely on only one example of life, i.e., life on Earth, which we refer to as the *N* = 1 problem [50], where one assumes all of the properties of life-based on a single observation of life. This idea then leads to another basic assumption in prebiotic chemistry research resulting from the current sample size of known life: Because all known life undergoes both replication and metabolism using specific biomolecules, the OoL necessarily utilizes the same biomolecules for the same purposes. Thus, finding prebiotically plausible mechanisms for such biomolecules to have been synthesized in early Earth geochemical systems, and their subsequent organization into supramolecular structures that accomplished replication and/or metabolism functions, is a relevant method to understand the OoL. This assumption is also extended in the search for ET life, which follows a teleological (directed) arc similar to the OoL of life as we know it. This line of thought then results in the idea that ET life (or signs of its existence) can be detected via biosignatures by detecting the same biomolecules found in life on Earth. This methodology is not unreasonable, especially in understanding the terrestrial OoL, as we only know one instance of life on Earth. However, this methodology overlooks a few possibilities important for the search for ET, which we will review in more depth:

(a) A “messy” chemical state is the default state of any prebiotic chemical space on a given planetary body of astrobiological interest (including primitive Earth). Such a messy prebiotic chemical space provides the starting material for prebiotic chemistry to happen, is likely different from planet to planet, is impossible to simulate completely precisely in the laboratory, and may not even be necessarily reproducible on the same planet.
(b) Non-biological chemicals could have played a role during the OoL, which may have produced life with a molecular makeup different from life on Earth.
(c) The role of contingency could shape life differently from life as we know it on Earth due to differing planetary conditions and “messy” prebiotic chemical spaces.

## 3. The “Messy” State of Prebiotic Chemistry

In one of our co-author’s early papers, the concept of ‘messy chemistry’ was introduced as the default mode of prebiotic chemistry, suggesting that the complex chemical milieu of early Earth was too intricate to have immediately led to the emergence and accumulation of modern biomolecules. Such complexity may differ from one local geoenvironment to another [51], where the environmental parameters (e.g., temperature, pressure, light intensity, time scale, minerals, etc.) could define what prebiotic chemicals may be useful for the OoL. Nevertheless, this “messy” chemistry concept was developed in response to the observation that most prebiotic chemistry experiments focus on the origin and reactions of primitive biomolecules to explain the OoL and suggested that a broader chemical scope may be necessary to fully understand the chemical OoL.

For instance, some researchers demonstrate step-by-step organic chemical synthesis to create desired biomolecules (e.g., RNA) [52] from prebiotic molecules, while others emphasize specific prebiotic molecules (e.g., tricarboxylic acids) to showcase metabolism-like prebiotic systems [53,54]. These works are truly exceptional from both fundamental and experimental perspective. However, they may not fully elucidate the exact mechanism of OoL in isolation (whether this is the goal of such work should, of course, be considered, as some OoL research focuses on elucidating possibilities that could have led to terrestrial life rather than the exact steps that led to terrestrial life), as they do not necessarily take into account the default state of prebiotic chemical spaces, which is essentially ‘messy’. Of course, such experiments are also limited by the available technology in our modern labs, which still cannot completely analyze extremely complex mixtures quantitatively [55], which suggests that some of the possible oversights in the current OoL research field are a result of technological limitations rather than ideological ones. However, such a discussion is out of the scope of this paper, as one can only use what current resources are available (while waiting for further technological development in the future).

Examples illustrating the “messiness” of prebiotic chemical space include organic compounds within carbonaceous chondrites or products of Miller–Urey-type chemistries [56], hydrogen cyanide (HCN) polymerization [57], formose reactions, hydrothermal vent chemistries [58,59], and more [39,60]. For instance, it is estimated that just a few milligrams of the Murchison meteorite, a carbonaceous chondrite frequently employed as a reference to the prebiotic plausibility of organic compounds, contain approximately 14,000 to 50,000 unique non-biomolecules, alongside minuscule (but non-zero) quantities of biomolecules [61]. Furthermore, even though some meteoritic biomolecules can be present at concentrations exceeding 1 µg/g (e.g., amino acids), the wider chemical classes they belong to contain both forms found in life on Earth and those that are not [62,63]. As such, the best-characterized extraterrestrial environments (meteorites) are incredibly messy.

Given the abundance of non-biological organic chemicals within these prebiotic chemistry spaces, at first glance, it may have been improbable that scarce prebiotic biomolecules would exhibit highly efficient, unique, and selective reactivity sufficient to initiate the initial stages of chemical evolution (e.g., [64]). Consequently, we may assume a similar selective reactivity between primitive biomolecules and non-biomolecules. Although this has not been irrefutably proven or disproven for all such combinations/mixtures of prebiotic chemicals, some specific studies show selective reactivity of biomolecules over non-biomolecules (or non-canonical biomolecules) or vice versa [65,66]. However, these laboratory demonstrations use fairly simple mixtures that may not have represented the actuality of the prebiotic chemical milieu. It therefore follows that it would have been statistically more likely that the significantly more abundant non-biomolecules served as the primary chemical actors during the OoL. Moreover, there is now suspicion that some biomolecules in contemporary life are the result of more recent evolutionary innovations [67,68,69] and are not directly derived from Earth’s prebiotic era, implying that these biomolecules were synthesized and/or accumulated *after* “life” had reached a certain level of development. Thus, understanding which biomolecules were likely of prebiotic origin and which ones more recently emerged (one goal of OoL research) would be helpful information on the relevance of certain biosignatures in future life detection missions. However, until then, due to their sheer number, non-biomolecules in the messy prebiotic Earth are one avenue that must be focused on, more specifically concerning their role at the OoL.

## 4. Non-Biological Chemicals and Their Prebiotic Chemistry During the OoL

The issues we have highlighted above regarding focusing only on prebiotic biomolecules as biosignatures have prompted us to reconsider what the essential ingredient(s) and recipe(s) were in the context of the OoL, if one *disregards* biomolecules as the primary actors. One solution is to consider plausible prebiotic non-biomolecules existing within a primitive chemical space that could react to form higher-ordered structures, such as polymers, aggregates, compartments, multilayers, and other phase-separated systems, and observe the outcomes. For example, we have previously written a piece about this topic [30] in which we emphasized the role of non-biomolecules at the OoL and considered that the earliest stages of the OoL were opportunistic and chemical reactions proceeded according to simple thermodynamics and kinetics rather than in pursuit of specific functions [70,71]. This opportunism allowed any compound with a physicochemical advantage (e.g., possessing moieties capable of facile polymerization) to initiate and drive chemical evolution. These compounds could then preferentially synthesize or assemble into higher-ordered structures, which could further self-organize and develop functionalities (e.g., proto-replication and metabolism, cellularization, etc.), thus guiding primitive chemistry to a point where Darwinian-like evolution can take over.

One such plausible OoL model, which the authors of this review have contributed to, focuses on the relevance of prebiotically available compounds capable of forming ester bonds, i.e., compounds that can produce polyesters. We argue that these compounds (e.g., lactones, lactams, hydroxy acids (HAs), etc.), all of which were potentially available on early Earth or meteorites (e.g., [72,73,74]), hold a thermodynamic advantage over their biological counterparts (e.g., amino acids) in terms of prebiotic polymerization through dehydration synthesis [75]. For example, using simple dehydration, ester-forming monomers can form feasible swathes of higher-order structures, including long and heterogeneous polyesters [29]. We achieved this result by subjecting several different free HA monomers (both in isolation and as parts of HA mixtures) to a single dehydration cycle, simulating the drying of primitive water bodies, to produce combinatorial polymer libraries. Some of these combinatorial polymer libraries exhibited microdroplet assembly propensity when rehydrated in an aqueous solution [76]; the assembled microdroplets exhibited some characteristics similar to those that are expected of protocells [77,78], primitive cellular-like compartments that eventually evolved into modern cells [79]. For instance, select polyester microdroplets preferentially segregated small organic dyes, a protein, a fluorescently labeled RNA, and salt cations [80], suggesting the potential of polyester microdroplets to contribute to primitive segregation and compartmentalization functions (which itself is an important “life-like” property for both analyte preservation and reaction driving [81,82]). Thus, these experimental demonstrations show that simple prebiotically available non-biomolecules can react to produce higher-order structures exhibiting potential life-like properties, suggesting their plausible participation at the OoL and thus providing an alternative non-biological view of the OoL. Other authors have also indicated and demonstrated similar concepts through different systems, such as depsipeptides [64], non-canonical sugars of RNA, PNA, etc. [83,84], suggesting the wider plausibility that non-biomolecules were active, possibly even essential, players at the OoL.

However, while experimentally exploring the role of non-biomolecular compounds at the OoL on Earth is valid, there is still no general framework that links prebiotic chemistry with the directional flow of contingency that can lead toward Darwinian-like life on and off Earth. In the next section, we synthesize concepts from both contingency and non-biomolecular OoL theories to formalize this relationship, which may lead to greater exploration first of non-biomolecules in prebiotic chemistry and later as possible biosignatures [85,86].

## 5. The Role of Contingencies during the OoL (on and off Earth) Linking Non-Biomolecular Compounds

**Figure 2:**
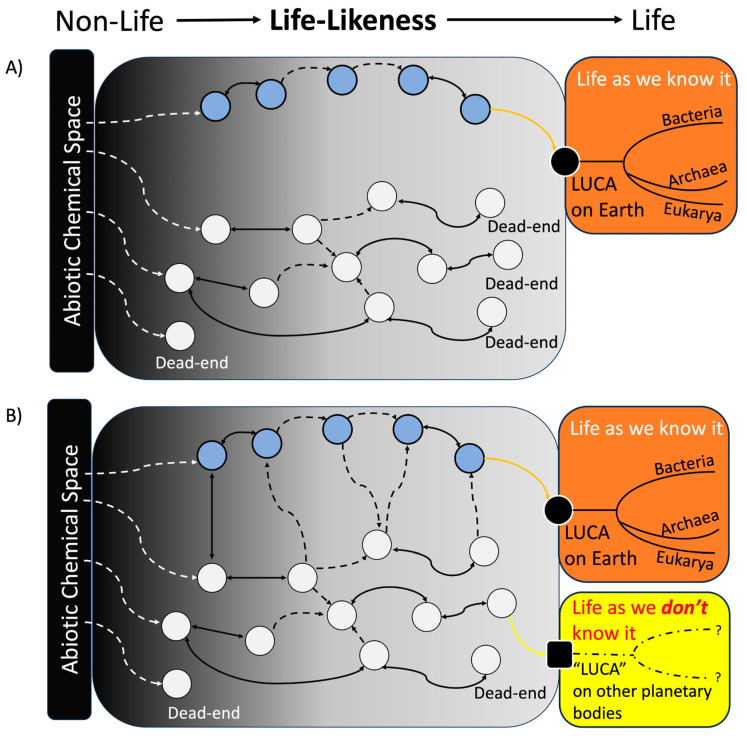
The model discussed here allows us to speculate about the initial formation of life as we know it (such as terrestrial life), or, life as we *don’t* know it, i.e., ET life. An arbitrary spectrum between non-life and life spans the diagram, indicating the transition and varying characteristics from abiotic to biotic forms. The starting point of life is arbitrarily termed the Last Universal Common Ancestor (LUCA) for simplicity. (**Panel A**) shows a deterministic origin of life (OoL) view, assuming a direct link between abiotic chemical space and biochemistry towards LUCA and, by extension, life as we know it on Earth. (**Panel B**) presents a contingency view, illustrating the numerous alternative paths that could have led to the OoL on Earth or on other planetary bodies, where a different type of “LUCA” could have appeared, representing life as we *don’t* know it. Below, we provide a legend detailing what each aspect of the model signifies.

In the previous two sections, we highlighted that the default state of prebiotic chemistry is “messy” and that non-biomolecules from prebiotic chemical spaces could have initiated or played a role during the OoL on Earth (and possibly elsewhere) due to their opportunistic physical/chemical properties and/or for statistical reasons. What remains unclear are the possible paths these prebiotic chemicals might have taken to reach a stage where a form of evolution, akin to Darwinian, could emerge and eventually lead to life. We have mentioned earlier that most OoL models assume a direct route from prebiotic chemistry to biological life focusing on biomolecules, i.e., the deterministic OoL (shown as the blue circles in Figure 2A). A Gould-like contingency narrative can help explore the numerous potential paths that primitive non-biomolecules might have taken, leading to an origin of life event, limited only by our current understanding and speculation (Figure 2B). Such an OoL event may have happened in the early Earth, leading to life as we know it, and may also have also occurred, or is occurring, on ET astrobiologically relevant planetary bodies of interest.

- **Black circle** = arbitrary LUCA on Earth, eventually leading to the terrestrial life tree (orange box).
- **Black square** = arbitrary “LUCA” on another planetary body, which eventually follows its own unique “phylogenic” path towards ET life (yellow box).
- **Blue circles** = deterministic chemical states (CS) inspired by and leading to modern biochemistry, such as an RNA world, a metabolism first world, etc., which assumes a direct history between prebiotic chemistry and modern biology.
- **White circles** = the diverse potential CS that may not follow a deterministic path toward life. These CS are composed of a blend of biomolecules and non-biomolecules, likely dominated in abundance by non-biomolecules originating from the abiotic chemical space, encompassing plausible abiotic chemicals that existed on primitive Earth or other early-planetary bodies. Contingency (by geochemical “selection”) propels CS further towards different areas of the “life-likeness” spectrum (i.e., non-life, somewhat life-like, and life) towards a more “life-like” state (defined from a terrestrial perspective). More advanced “life-like” CS can move forward along this spectrum by manipulating their environment, potentially metabolizing and replicating through the use of novel molecules not found in the abiotic chemical space (i.e., autotrophy), all while still undergoing “selection”, advancing their status to become arbitrary “LUCA”. At any given point, any CS can reach a dead-end, meaning such a CS cannot progress to any form of life.
- **White dashed arrows** = the process for a CS to emerge, which is derived directly from a particular abiotic chemical space, i.e., all components within the CS are solely chemicals within the abiotic chemical space of any geological niches on early Earth or other planetary bodies.
- **Double-headed solid black arrows** = a transition from a CS to another CS. These black arrows are double-headed, which suggests that a CS can return to a former state through, for example, the degradation of certain components.
- **Dashed black arrows** = the mixing of two or more CS (the two or more circles at the origin of the arrows) to form a novel CS composed of a mixture of components of the initial CS (the one circle at the destination of the arrows). These arrows are single-headed, as de-mixing may not result in recovery of identical initial CS as before; however, de-mixing (not shown) could result in one CS splitting into two novel CS. The mixing can result from the mixing of two or more biomolecular CS (blue circles), some “non-biomolecular” CS (white circle) with some biomolecular CS, or two or more non-biomolecular CS. For example, mixing of a non-biomolecular CS with a biomolecular CS can result in a new CS where the components of the initial non-biomolecular CS drive some type of novel reaction amongst the components of the initial biomolecular CS, ultimately leading to the emergence of a more “life-like” biomolecular CS that is “scaffolded” by some non-biomolecular process or chemical(s).
- **Orange and yellow arrows** = the progression of a CS to a LUCA. These arrows are single-headed, indicating the fact that once LUCA emerges, which is defined as “life”, it cannot reversibly convert back to a “non-life” CS state (as “life” by definition must either be able to sustain its own life, or undergo extinction (see below for further explanation).

The model’s description, underlying assumption, and caveats are detailed in the main text below.

A model linking both contingency and non-biomolecular OoL requires several definitions (Figure 2). First, the abiotic chemical space encompasses plausible abiotic chemicals that existed on primitive Earth or other early-planetary bodies (such as the Noachian Mars or the early moons of the gas giant planets). The precise composition and quantities of actually available abiotic chemicals strongly hinge on planetary-scale geological processes. This reliance results in variations based on location, such as within the atmosphere, at the interface between early oceans and land and in the atmosphere and bodies of water, or potentially even in deep-sea hydrothermal vents, provided geological conditions allow such geological features on a specific celestial body. Moreover, the composition and quantities of available abiotic chemicals may undergo changes within these specific locations due to geological changes with time. For example, the silicate-carbonate cycle (on early Earth) is crucial in determining the amount of CO_2_ in the atmosphere. This, in turn, can trigger feedback loops in determining the overall climatic conditions during the early stages of a celestial body’s development, thus possibly changing the composition and quantities of available abiotic chemicals [87].

Second, we define a chemical state (CS) as a blend of chemical composition and structures possibly therein within a specific location at a given point in time (circles of blue and white) (Figure 2). For example, initial CSs that emerged (indicated by white dashed arrows) would have contained only chemicals found in the abiotic chemical space (e.g., chemicals available on prebiotic Earth) and would have been less “life-like” along the “life-likeness” spectrum. This spectrum, which is arbitrarily defined at this point, could be a combination of several parameters. The definition of CS is made to be intentionally broad, as it is impossible to definitively determine the precise chemical state that emerges from a particular abiotic chemical space. Initial CSs could, for instance, include polymers that existed in the abiotic chemical space (such as macromolecular organic matter, which can be found in meteorite samples [88,89]) or even be an autocatalytic system of small molecules.

In the model shown in Figure 2, CSs can change in one of two ways. For example, CSs can change over time through modifications in chemical composition (solid black arrows, (<->)), i.e., changing environmental pressures and/or geochemical reactions, including (but not limited to) thermal- [85] and photo-destruction [86,87], dilution [88], hydrolysis [90], dehydration synthesis [90], etc. This type of CS transition is reversible due to the possibility of both the synthesis of new molecules from reactants in a particular CS, as well as the degradation of this new molecule, reverting to an identical composition as a previous CS.

Another mechanism of CS transition is through mixing with another CS from a different location (e.g., by micro-meteorite impact [91], or physical mixing of different CS caused by natural terrestrial phenomena, such as the ebb and rise of small water bodies). The dashed black arrows (--->) in Figure 2 indicate two or more CS mixing (circles at the origin of the arrows) to form a novel CS containing the composition of the initial CS (circles at the destination of the arrows). For example, a CS with a majority of non-biomolecules could produce an important catalyst or raw materials needed to transition a different CS containing a majority of biomolecules into a more life-like state.

As a CS progresses towards greater life-likeness (i.e., from left to right in the figure), its constituents would begin to engage in reactions (and interactions) that require reactants not originally present in the abiotic chemical space (for example, macrostructures such as complex polymers). For a more “life-like” CS, these reactants could be produced internally or sourced from another CS, indicating that the system has become autonomous and less reliant on the initial abiotic chemical supply. This suggests that the CS has become more autotrophic, but it may still rely on interaction with other CSs to achieve autotrophy).

More life-like CSs may also result in the maintenance of a more limited chemical composition that contains only those chemicals completely needed for its own survival. This is in contrast to emergent CS, which may be quite diverse due to the complexity of the abiotic chemical space. A more life-like CS might achieve greater efficiency in its reactions by concentrating only the essential reactants. In some cases, a CS that is fairly life-like may reach a “dead-end state” where it cannot progress further towards a further life-like state. Such dead-ends can even happen even at the initial stages of the CS. However, despite advanced CSs’ incapability to move, they could revert to a previous, less “life-like” state, as indicated by a double-headed solid black arrow (<->). The reasons for this stagnation and possible backtracking may include degradation, geochemical bottlenecks, missing crucial chemical reactions or products produced by itself or by another CS, or an inability to exhibit certain life-like properties, such as stability, necessary for persistence. An unpredictable global or local scale event could also cause a CS to be wiped out en masse during its course toward life. It is also plausible that many CSs that contained a majority of non-biomolecules ended up in a “dead-end” state, as evidenced by the importance of biomolecules in extant biology (Figure 2A). Eventually, there is historical tractability of CSs on Earth (blue circles) that eventually led from abiotic chemistry (perhaps with the help of various CSs containing both biological and non-biological molecules) towards the first life, i.e., LUCA (dark circle), which eventually leads to the tree of life of Earth (orange panel, right side of Figure 2B).

However, perhaps the history of CSs on Earth that results in a CS prior to LUCA requires inputs from other CSs that themselves can never result in life as we know it, e.g., those composed of non-biomolecules that terminate to a dead-end state before reaching life (Figure 2B) [25]. In this way, rather than viewing the emergence of life as a direct path (blue circles) of subsequent events starting from abiotic chemical space and ending up at LUCA (Figure 2A), one must also consider orthogonal chemical systems, states, reactions, and products, including those involving so-called non-biomolecules, that may have themselves contingently and directly impacted the initial emergence of life on Earth.

The model introduces an alternative perspective to the deterministic OoL view (Figure 2A). For instance, as shown in Figure 2B, non-biomolecular CS could have “scaffolded” the emergence of biomolecular CS (shown by the arrows linking the white and blue circles), moving towards LUCA and life as we know it (depicted in the orange box). Conversely, depending on each CS’s contingent path, which is highly influenced by environmental and geological pressures, non-biomolecular CSs could potentially traverse the spectrum of “life-likeness”, giving rise to alternative “LUCA” and (possibly) branching to become alternative life (depicted in the yellow box) that is distinct from life as we know it. Such a process could be plausible on another planetary body (e.g., within the solar system or an exoplanet such as Kepler-452b [92]). Moreover, such alternative life forms might not be detectable through biomolecule-based biosignatures but would depend on agnostic biosignatures [86]. Examples of such “bio”signatures are unique molecular frequency patterns that are different from their abiotic chemical space [85], higher enantiomeric excess of compounds, or even the signs of catalytic activities [93].

**Figure 3:**
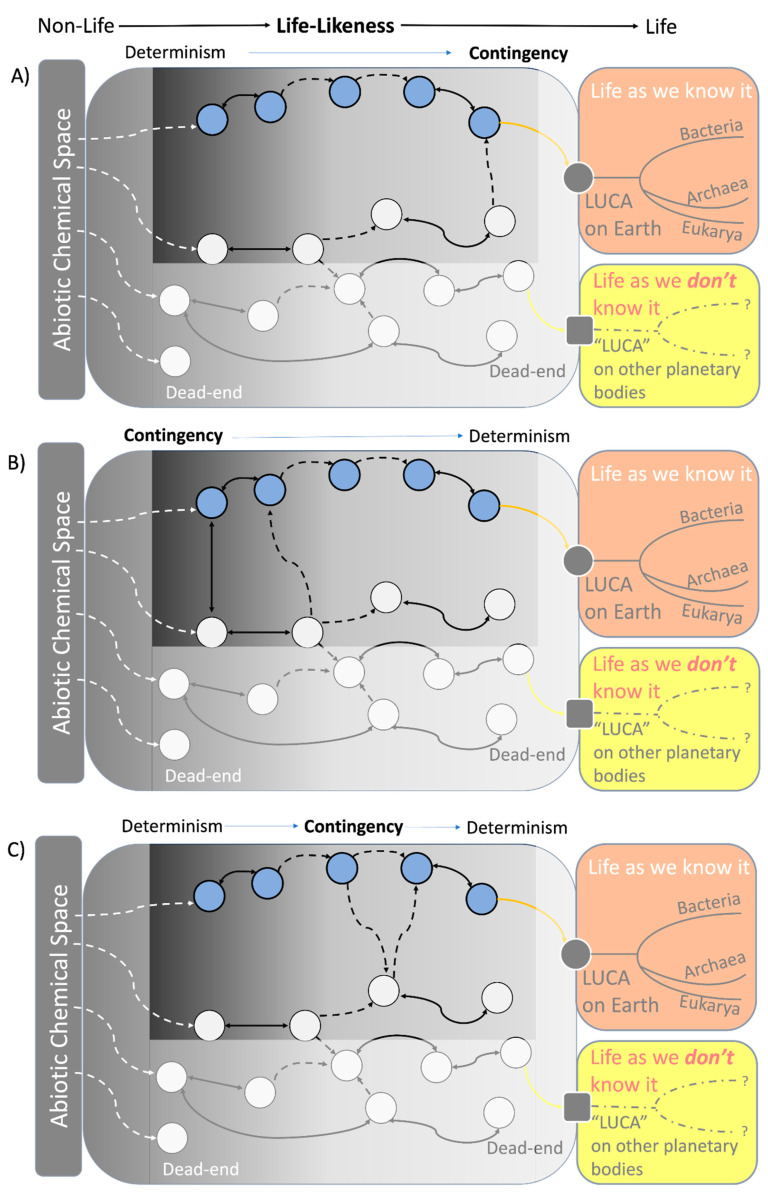
A spectrum between contingency and determinism (or a mix of both) is likely to exist. The highlighted plane in (**Panel A**) shows that determinism can occur early on during a CS non-life phase and become more contingent as it progresses toward life. Conversely, the reverse is also feasible, as depicted in the highlighted plane of (**Panel B**), whereas the highlighted plane of (**Panel C**) illustrates that intermixing of both contingency and determinism can happen between a deterministic starting point and an endpoint of a CS before it becomes LUCA. Alternatively, such intermixing can also happen between a contingent starting point and an endpoint of a CS (diagram not shown).

On a separate note, the binary distinction between deterministic OoL (shown in Figure 2A) and contingent OoL (shown in Figure 2B) could present a false dichotomy by presenting two options as the only possible choices, ignoring other viable alternatives. Such alternatives include the likelihood of a spectrum between determinism and contingency (Figure 3A,B), or an intermixing of both (Figure 3C). This phenomenon may not only apply to the OoL on Earth but also to the OoL of ET elsewhere.

However, we note that our model makes several assumptions worth pointing out, for instance:

**Note 1**: The arbitrary term LUCA was used as a point depicting the beginning of Darwinian-like evolution, but the concept of LUCA remains unclear in the field; for example, LUCA could either be a living entity that acquired the full ability of transcription-translation mechanisms [94] or one derived from progenotes [95,96]. The latter is not depicted in Figure 2 for simplicity. Similarly, the definition of terms we use to describe life in this paper, such as ‘life as we know it’ and ‘life as we don’t know it’, is not discussed here, as it is well covered elsewhere [97].
**Note 2**: Though not explicitly shown in the model, the arrival of unique elements or organic compounds via by means of comets and meteorites [98] could have occurred during meteoritic bombardment periods, which were was common on Early Earth [99]. These additions might have influenced the available abiotic chemical space, potentially affecting the progression of “life”, and the existence of more “life-like” CSs at different times. This could also be applied to ET life cases. In this case, more emergent CSs could have emerged from the altered abiotic chemical space, while more “life-like” CSs could incorporate some aspects of the modified abiotic chemical space to access novel chemicals, chemistries, reactions, and functions. This concept simplifies to a larger abiotic chemical space, which can be seen as encompassing components from both the Earth’s abiotic chemical space and that introduced by comets and meteorites.

**Note 3**: Although not mentioned explicitly, emergent polymers from the initial abiotic chemical space with early catalytic abilities could have altered the reaction topography within its own chemical space, leading to the formation of new CSs. These new CSs either enhance or hinder the life-like properties of a particular CS. Additionally, the formation of these new CSs could also be maintained by auto-catalytic reactions, potentially giving rise to a where new functional chemistries and reducing the dependence on input from other CSs for persistence.
**Note 4**: Terrestrial life may be an outlier, and existence of ET life remains improbable (but not impossible), as we have not observed a single instance of ET life. Several factors contribute to the absence of signs of ET life, possibly because such life never developed. Firstly, chemistries that lead to the OoL (as well as the chemistries of life) must be viable for a long period of time from the onset of planetary formation due to the timescales of non-catalyzed chemical reactions [100]. Secondly, early OoL events (and life itself) occurring on a particular planet must remain persistent, i.e., keeping pace and changing to survive in the ever-changing environmental and geochemical settings by developing replication and fine-tuning metabolism and protection to avoid extinction [101]. Life on Earth has adapted and survived for ~3–3.5 billion years despite numerous extinction events. Hence, if any of the requirements for OoL off Earth are not met, we will never observe extant ET life, either due to early extinction of early life or failure to fend off its last planetary extinction threat. In either case, the planet would remain barren of life.

## 6. Caveats of the Contingency and Non-Biomolecular OoL Model

The reader should bear in mind this model only serves as a possible narrative formally linking contingency with the role of non-biomolecules at the OoL based on concepts discussed therein without imposing specific theories (e.g., non-equilibrium thermodynamics [102], network theory [103], systems chemistry [104], etc.) and higher-level abstractions. The omitted higher-level abstractions include exploring an ecology-like global system exhibiting characteristics akin to present-day life on Earth, i.e., their direct or indirect interactions and influence on the global environment and climate that, in turn, influences the overall geosphere and biosphere of their planet. These omissions were made deliberately to offer simplicity and generality to the initial model so that any theory can be linked with and applied to the model at a future date, as it will be crucial to study how a group of CSs moving towards life-likeness are interlaced with the theories mentioned above and the ecological system at a planetary scale. Nevertheless, the model shown above has several caveats requiring further study, for instance:

(a) We did not specify the specific chemical, geological, and physical constraints necessary for conditions leading to life in the model because such constraints are not necessarily fixed and are bound to be updated with new data and modeling. Nevertheless, the emergence of any CS, like any prebiotic system, is driven and governed by constraints via its primitive environment parameters (pH, temperature, etc.) [51]. Early CSs are constrained by the rules of chemistry, which are governed by thermodynamics (before kinetic control) and also geology, i.e., geological niches present on early Earth (e.g., hydrothermal vents, evaporating ponds, etc.). Such constraints act as “selective” pressures on CSs and can fluctuate from time to time, either in short or longer durations. As CSs evolve to become more complex and functional, resembling life forms (as illustrated in Figure 2), the imperative to adapt to such constraints for survival intensifies. Sudden changing chemical and environmental constraints due to ambient geology, for example, temperature rising because of increasing atmospheric CO_2_ from volcanic events, may be too harsh on advanced CSs made up of macromolecules and could lead to thermal degradation. For example, RNA and polyesters within a particular CS in water are prone to accelerated hydrolysis when heat is added [105,106]. Alternatively, oceans getting cooler due to tectonic events, i.e., the movement of Earth’s primordial continental plates [107] allowing movement of water between them, could slow down the chemical reactions that make certain CSs. How these constraints act on a particular CS is of particular interest and helps evaluate their plausibility for survival and innovation (which may come when an (advanced) CS has the ability to create solutions for a problem likely via some replication system).
(b) The presence and composition of the actual chemicals available within any abiotic chemical space is contingent upon the prevailing local and global geochemistry of a specific celestial body of astrobiological significance, e.g., early Earth or Noachian Mars. This likely will change from time to time (e.g., [108,109,110]) during the prebiotic epoch and across geological timescales, and hence primitive chemical systems will not be provided with continuous supplies of *all* organic chemicals *all* of the time; the supply of critical organic compounds would have implications on both the ability to generate emergent CS and on the identity of the emergent CS (it may be that no single emergent CS is identical to any other one). Global or local perturbating geological conditions provide further screening on the heterogeneous emergent CS [51], for example, through degradation or seclusion, that can eliminate some or all emergent CS from existence. This can lead to the preservation of the “fittest” CS, in terms of the persistence, while “functional” CS—those with abilities important for progressing towards a more “life-like” state, such as autocatalytic properties—may be expelled during such perturbations if they are not fit for persistence. A “functional” CS may be lost altogether at some point in time, although the function may be deemed useful towards achieving more “life-like” states. Hence, how would the system recover such a loss? Can the same lost “functional” CS be produced again if the chemical and geological conditions revert to the original state (e.g., the reversal of a snowball Earth-like event to a warm Earth-like one [111]) that produced the lost “functional” CS in the first place (it may never revert in the end)? Or can the system produce another CS that can perform the same (or a similar) function as the lost “functional” CS, simply with different components? This would, of course, depend on the function, and whether that function must necessarily be achieved in one way, or whether there are multiple ways to achieve that function. As the introduced model consists of a multitude of emergent CSs, which theoretically are constantly being produced (from the abiotic chemical space) and degraded, what are the probabilities of recovery of a lost “functional” CS if emergent CS may never be identical to any other one (and if more than one CS is required to lead to another resulting CS)? This is where we anticipate determinism coming into play, where the lost functionality can be possibly re-gained through a different pathway towards the formation and/or composition of a CS.
(c) Without a replication system, Darwinian-like evolution is not possible, and the long-term existence of any life-like CSs edging closer to life in the spectrum of “life-likeness” is unsustainable. This is because the formation and continuity of CSs depend on the constant availability of the same chemical sources that originally produced them. As mentioned before, the constant changes in geological conditions on early Earth could have caused chemical fluctuations that led to the rapid disappearance of a multitude of CSs, both emergent and those that are more “life-like”. If any CS indeed had a hand in the initial development of a living system, it would likely have required the ability to replicate informationally at some point. This replication would allow the CS (or a similar CS produced later) to persist long enough to transfer its structures, functions, products, or reactions to the primitive living system, even amidst fluctuations and the loss of chemical availability (i.e., it must be able to be replicate, assuming that there is only one deterministic pathway to the emergence of life). This replication could have been through a genetic sequence system [112], a composomic informational system [113], a constant supply of the required materials (see above) through some type of auto-catalytic system [114], or some other type of not-yet-known replication system.
(d) We have shown the possibility that sequences of prebiological chemical states that eventually led to the emergence of arbitrary LUCA may have been “scaffolded” by non-biological compounds throughout history’s existence (Figure 2B). If the conclusion from [69] is true—that the inclusion of biological choice from chemical space of biomolecules bootstraps a CS towards producing biological products—then understanding when and how the processes involving non-biomolecules (if they were essential for the emergence of life) were removed from primitive biology is crucial and requires further investigation. For example, it has been shown that alpha hydroxy acid-charged-tRNAs can undergo in vitro translation that results in the synthesis of polyesters with modern biological translation machinery [115,116]. Does this mean that if alpha hydroxy acids and/or polyesters participated in primitive biology, that they were removed only after the emergence of the central dogma? Or, is this simply a biochemical coincidence? Were there other non-biological systems that participated in primitive biology that are now absent in modern biology? How do we know if there are?

## 7. Summary

Here, we review and synthesize contingency and non-biomolecular models of the OoL, suggesting that non-biomolecules may have played an important role in the OoL on Earth; by extension, non-biomolecules may thus play an important role in the origin of ET life. Therefore, we recommend considering such molecules in extraterrestrial life detection missions.

While we acknowledge some potential caveats of this model, the prebiotic chemical space is vast, and the applicability of non-biomolecules in the initial emergence of terrestrial life is certainly within reason, considering the sheer abundance of non-biomolecules at the early stages of life. Although this model mostly draws from lessons learned from investigating evolutionary biology and Earth’s OoL, it is also applicable to ET life, as there is also no specific reason for or against the belief that ET would be identical to modern biology [98]. Thus, we hope that by combining contingency with non-biomolecular OoL theories, such as that herein, inspires those in ET life detection to further consider searches for relevant non-biomolecules (such as alpha hydroxy acids and polyesters, PNA, etc.) that may indicate life or have participated in the OoL.

## Figures and Tables

**Table 1:** Terms and their definitions used in this paper.

Term	Definition	Reference
Abiotic Chemistry	Any chemistry involving non-living or inanimate matter.	[24]
Prebiotic Chemistry	A subset of abiotic chemistry that has led to life or chemistry that happened during the OoL.	[24,25]
Biomolecules	Molecules that make up the structure and function of living organisms on Earth.	[26]
Non-Biomolecules	Refers to prebiotic organic molecules, which are not directly employed in biochemistry or serve different roles to their biochemical functions. An example of non-biomolecules are alpha hydroxy acids. These compounds can polymerize prebiotically to become prebiotic polyesters. While some alpha hydroxy acids (e.g., lactic acid, malic acid, etc.) are considered to be biomolecular compounds in the broad sense, with roles in respiration, inflammation regulation, and molecular signaling [27,28], they are considered non-biomolecules in the prebiotic context. This is because, in prebiotic chemistry, these compounds are involved in polymerization [29], a function not used by living organisms for these compounds. Additionally, within the paper, it is important to distinguish between the terms ‘biomolecules’ and ‘non-biomolecules’ for clarity. Even if some molecules transition from prebiotic to biological roles, the term ‘non-biomolecules’ are *not* reclassified to ‘biomolecules’. However, outside this paper’s scope, biomolecules could include those that originally were non-biomolecules but became integral to life on Earth or elsewhere.	[30] and further updated in this paper
Building Blocks	Refers to the fundamental chemical compounds, biomolecular and/or non-biomolecular, essential for the formation and function of life. This concept applies to terrestial life forms [31] and hypothetical or theoretical forms of extraterrestrial (ET) life.	[31] and this paper
Terrestrial-biochemistries	Refers to the biochemical processes and molecular compositions associated with life as we know it on Earth.	[32]
Xeno-biochemistries	Refers to “bio”chemical processes and ”bio”molecular compositions associated with ET life forms that could exist in environments different from those on Earth. In this paper, Xeno-biochemistry could, in principle, accommodate both biomolecules and/or non-biomolecules within its functionality and form. The word ‘Xeno-biochemistries’ carries the connotation of ‘xeno-biology’. However, other definitions of xeno-biology include the design and engineering of new life forms by humans using technology [33]. For the sake of simplicity, we will equate the ET definition mentioned above with xeno-biology.	This paper
